# A Dynamical Model of Oocyte Maturation Unveils Precisely Orchestrated Meiotic Decisions

**DOI:** 10.1371/journal.pcbi.1002329

**Published:** 2012-01-05

**Authors:** Benjamin Pfeuty, Jean-Francois Bodart, Ralf Blossey, Marc Lefranc

**Affiliations:** 1 Laboratoire de Physique des Lasers, Atomes, et Molécules, CNRS, UMR8523, Université Lille 1 Sciences et Technologies, Villeneuve d'Ascq, France; 2 Institut de Recherche Interdisplinaire, CNRS, USR3078, Université Lille 1 Sciences et Technologies, Villeneuve d'Ascq, France; 3 Laboratoire de Régulation des Signaux de Division, EA 4479, Université Lille 1 Sciences et Technologies, Villeneuve d'Ascq, France; Princeton University, United States of America

## Abstract

Maturation of vertebrate oocytes into haploid gametes relies on two consecutive meioses without intervening DNA replication. The temporal sequence of cellular transitions driving eggs from G2 arrest to meiosis I (MI) and then to meiosis II (MII) is controlled by the interplay between cyclin-dependent and mitogen-activated protein kinases. In this paper, we propose a dynamical model of the molecular network that orchestrates maturation of *Xenopus laevis* oocytes. Our model reproduces the core features of maturation progression, including the characteristic non-monotonous time course of cyclin-Cdks, and unveils the network design principles underlying a precise sequence of meiotic decisions, as captured by bifurcation and sensitivity analyses. Firstly, a coherent and sharp meiotic resumption is triggered by the concerted action of positive feedback loops post-translationally activating cyclin-Cdks. Secondly, meiotic transition is driven by the dynamic antagonism between positive and negative feedback loops controlling cyclin turnover. Our findings reveal a highly modular network in which the coordination of distinct regulatory schemes ensures both reliable and flexible cell-cycle decisions.

## Author Summary

In the life cycle of sexual organisms, a specialized cell division -meiosis- reduces the number of chromosomes in gametes or spores while fertilization or mating restores the original number. The essential feature that distinguishes meiosis from mitosis (the usual division) is the succession of two rounds of division following a single DNA replication, as well as the arrest at the second division in the case of oocyte maturation. The fact that meiosis and mitosis are similar but different raises several interesting questions: What is the meiosis-specific dynamics of cell-cycle regulators? Are there mechanisms which guarantee the occurence of two and only two rounds of division despite the presence of intrinsic and extrinsic noises ? The study of a model of the molecular network that underlies the meiotic maturation process in *Xenopus* oocytes provides unexpected answers to these questions. On the one hand, the modular organization of this network ensures separate controls of the first and second divisions. On the other hand, regulatory synergies ensure that these two stages are precisely and reliably sequenced during meiosis. We conclude that cells have evolved a sophisticated regulatory network to achieve a robust, albeit flexible, meiotic dynamics.

## Introduction

**Figure 1:**
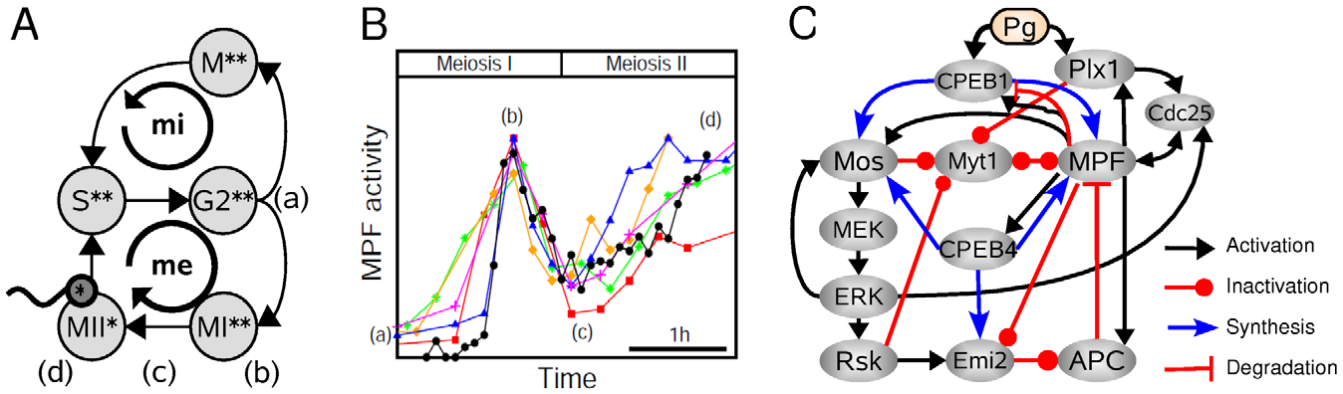
Temporal and structural organization of oocyte meiotic maturation. (**A**) State transitions during mitotic cycles (mi) and meiotic maturation (me). Haploid and diploid states are indicated by one or two asterisks, respectively. The four main stages of meiosis are (a) meiotic resumption following the progesterone pulse, (b) metaphase of the first meiosis, (c) meiotic transition and (d) metaphase arrest during the second meiosis. (**B**) The typical time course of MPF kinase activity during the maturation process of *Xenopus* oocytes where the four main stages (a–d) are indicated. Experimental data are from [54] (black circles), [7] (orange diamonds), [6] (blue triangles), [55] (magenta plus), [22] (green asterisks) and [13] (red squares). The zeroes of the time axis have been calibrated and the MPF axis have been normalized to have the first peak of MPF activy occur at the same time and with the same amplitude for each time course. (**C**) Detailed representation of the network of translational and post-translational interactions regulating metazoan oocyte maturation. This network involves a tight and precise coupling between the MPF and MAPK pathways at multiple levels. See text for details.

The mitotic division cycle is the sequence of events by which a growing cell replicates all its components, including DNA, and divides them, after mitosis, into two nearly identical daughter cells [1]. Meiosis is an alternative mode of cell division in which a diploid cell undergoes two successive divisions without intervening DNA synthesis, to create haploid cells called gametes or spores [2]. In vertebrate species, for instance, meiosis occurs during oocyte maturation, which is initiated in response to an hormonal signal with the specificity that oocytes are thereafter arrested, usually at the metaphase stage of MII, awaiting fertilization [3]. Meiotic maturation shares with mitosis many morphological events, such as metaphase and anaphase, as well as regulators such as the cyclin B-Cdk1, known as the M-phase promoting factor (MPF). However, it also involves a unique sequence of decision steps - meiotic resumption, transition and arrest - which clearly diverges from the mitotic one (Fig. 1A). Investigating the regulation of meiotic maturation is therefore an opportune strategy to understand the remarkable plasticity of the cell cycle, which unfolds a diversity of decision patterns at different stages of multicellular development.

The specific decision pattern of the oocyte meiotic maturation is intimately linked to the tightly controlled temporal dynamics of MPF (Fig. 1B). The rise and the first peak of MPF activity triggers germinal vesicle break down (GVBD) and entry into MI. The transition from MI to MII is typified by an unusual partial decrease of MPF activity followed by an increase and stabilization at a plateau level associated with metaphase II arrest in *Xenopus* oocytes. The time course of MPF is shaped by a complex web of interaction with other cell-cycle regulators. At the first arrest of *Xenopus* oocyte in a G2-like state, MPF kinase is stored in an inactive state called pre-MPF in which, among the five isoforms of cyclin B described in this animal model, only cyclin B2 and B5 are found associated to Cdk1 [4]. As during mitosis, MPF activity is primarily regulated by its interaction with a dual protein-phosphatase (Cdc25), a cyclin-dependent kinase inhibitor (Myt1) and the anaphase promoting complex (APC). During meiotic maturation, this module is supplemented with a layer of control which involves the MAPK (Mitogen Activated Protein Kinase)/ERK(Extracellular Regulated Kinase) pathway, whose main upstream and output components in the context of meiotic maturation are proteins Mos and Rsk, respectively. These components of the MAPK pathway are involved not only in meiotic spindle morphogenesis during oocyte maturation [5] but also at several decision points of the oocyte maturation process including meiotic resumption (G2/MI), meiotic transition (MI/MII) and maintenance of metaphase II arrest [6]–[8]. A key advance was to identify Rsk-mediated phosphorylation of APC inhibitor Emi2 as leading to MPF reaccumulation at the MI/MII transition [9]–[11]. In turn, MPF tightly controls phosphorylated levels of Mos [12] or Emi2 [13].

Two decades of experimental studies have thus documented manifold levels of interaction between the MPF and Mos/MAPK pathways, whose respective roles in various decision stages of maturation remain difficult to disentangle. In an attempt to clarify the interactions between both pathways, we use a modeling approach which has already been harnessed to gain insight into cell cycle control during animal development, as with the syncytial mitotic cycles in Drosophila embryos [14], fertilization process in mammals [15], the oocyte maturation initiation switch [16] but not yet for the whole oocyte meiotic maturation process. This approach has been remarkably successful, not only to check whether known molecular interactions can explain observed contextual and functional cell-cycle behaviors, but also to uncover the design principles of the molecular network in terms of feedback and feedforward topology [17]–[19]. Our modeling effort will thus be devoted to address two complementary issues: Are documented interactions between MPF and MAPK pathways necessary and sufficient to account for the observed properties of meiotic maturation? What are the network design principles that robustly enforce the progression of cells through a specific sequence of meiotic decisions?

To answer these questions, we first build a computational model that incorporates the major signaling pathways involved in the meiotic maturation of *Xenopus laevis* oocytes. Appropriate parameterization of the model allows us to reproduce the temporal dynamics of MPF and of other key regulators when the oocyte progresses from meiotic entry to metaphase II arrest. The dynamical mechanisms underlying these transitions are further analyzed using bifurcation analysis, which unmasks the existence of two main positive-feedback systems in addition to the core negative-feedback loop along which MPF represses itself by upregulating its own inhibitor, the anaphase promoting complex (APC). Remarkably, the architecture of these two subcircuits is unambiguously identified using a parameter sensitivity analysis, which reveals that they independently regulate meiotic resumption on the one hand, and meiotic transition on the other hand. The significance of the model is further assessed by simulating how alteration of the underlying molecular network using chemical manipulations or antisense strategies may induce maturation defects including initiation delays [8] or failures to transit from first to second meiosis [4], [6], [7], [10], [20]. Revisiting the relation between topology and dynamics in the maturation regulatory network leads us to identify and discuss the design principles that underlie the complex and reliable decision sequence studied here, and which could apply in various other cellular contexts.

## Results

### Model for the meiotic maturation regulatory network

The interaction graph shown in Fig. 1C incorporates all molecular actors and interactions known to be involved during the cell-cycle progression from prophase I to metaphase II arrest. Following the basic principles of biochemical kinetics, we translate this graph into a set of ordinary differential equations (see Methods). The unknown rate constants of the model are estimated by fitting the qualitative model behavior to the available data, including the well-characterized temporal profile of MPF activity during the meiotic maturation (Fig. 1B) as well as the bistable behavior of the MAPK modules and the oscillatory dynamics of the MPF-APC module (Methods). It was found that the behaviors observed depend little on the specific parameter set chosen under these constraints. This global maturation regulatory network of Fig. 1C connects together functional modules that so far have been studied only separately: (i) the MPF autoamplification loop, which triggers G2/M transition; (ii) the MAPK phosphorylation cascade, which is characterized by specific upstream and downstream components during meiotic maturation and is tightly bound to the MPF autoamplification loop; (iii) the underlying CPEB-dependent translational network, which controls temporal expression during the maturation process. We describe below how these subcircuits function and how they interact.

#### MPF autoamplification loop

The autoamplification loop features post-translational modifications which together lead to a sharp increase of CDK1 kinase activity. During meiotic maturation, this loop relies on interactions between MPF, Myt1, Cdc25 and Plx1. The activity of MPF is downregulated by Myt1 kinases and upregulated by Cdc25 phosphatases. In turn, MPF phosphorylates Myt1 and Cdc25 [21] thereby inactivating and activating these targets, respectively. Another critical regulator in this loop is the Polo-like kinase *Xenopus* 1 (Plx1) which both activates Cdc25 and inactivates Myt1 whereas it is activated by MPF [22]. Plx1 is also one of the main mediators of progesterone-dependent activation of the auto-amplification loop at meiotic resumption [23]. The role of protein kinase A (PKA) [24] and of protein Ringo [25] in activating the auto-amplification loop in response to progesterone is neglected here because their effect is roughly similar to that of Plx1. After G2/M transition, MPF also activates APC by both upregulating APC activity and inactivating and destabilizing inhibitors of APC, including Emi2. As it does in mitosis, APC leads to cyclin B degradation.

#### MAPK pathway

The MAPK signaling cascade is a highly conserved module across species, which is activated in a variety of contexts. During meiotic maturation, its main activator is Mos and its main target is Rsk [26]. Activation of Mos above a threshold level triggers a phosphorylation cascade of MAP kinases characterized by a sharp all-or-none activation which is amplified by the feedback phosphorylation of Mos by ERK [27]–[29]. The activity of MAP kinases and of their main upstream and target components have been implicated in many control points of MPF activity. First, activated Mos and Rsk phosphorylate and inactivate Myt1, a negative regulator of MPF [30], [31], though the role of these interactions is still controversial [32]. Second, ERK also directly triggers Cdc25 phosphorylation and activation [33]. Third, Rsk recruits PP2A (assumed to be constantly available and therefore neglected in our model) to phosphorylate Emi2/Erp1, promoting its binding to APC and preventing its phosphorylation by MPF, thus stabilizing the protein. In turn, MPF also regulates MAPK by controlling not only Mos phosphorylation [12], but also its synthesis as discussed below.

#### CPEB-dependent translational control

Regulation of mRNA translation through the unmasking of dormant mRNA is a key mechanism to control temporal expression during the maturation process. Cytoplasmic polyadenylation element binding (CPEB) proteins activate translation of specific mRNAs during meiotic resumption (Mendez and Richter, 2001). Two proteins, CPEB1 and CPEB4, are sequentially involved in this process and are differentially regulated by specific kinases (Igea and Mendez, 2010). The model is based on a simplified description of interactions between CPEB and other proteins. On the one hand, we assume that post-translational control of CPEB1 activity by Aurora A depends on both progesterone and MPF, whereas degradation of CPEB1 is achieved by the interplay of MPF and APC [34]. On the other hand, the model takes into account two main translation waves instead of three [34], where CPEB1 leads to the polyadenylation of multiple RNAs, especially those of Mos, CPEB4 and cyclin B1 and B5, while CPEB4 preferentially drives the translation of cyclin B2 and Emi2 mRNAs.

### Meiotic maturation dynamics: bistability, non-monotonicity and reliability

A remarkable feature of oocyte meiotic maturation is that a basic hormonal signal (a pulse-like or constant exposure of progesterone) induces a complex non-monotonous MPF activity profile (see Fig. 1B). The term *non-monotonous* refers to the fact that MPF activity does not continously increase or decrease during maturation but falls rapidly after rising to a first peak, before increasing again toward a plateau. In this section, we investigate the network dynamical properties underlying this sophisticated temporal profile of MPF activity, which drives the sequence of meiotic decisions from resumption to transition and arrest.

**Figure 2:**
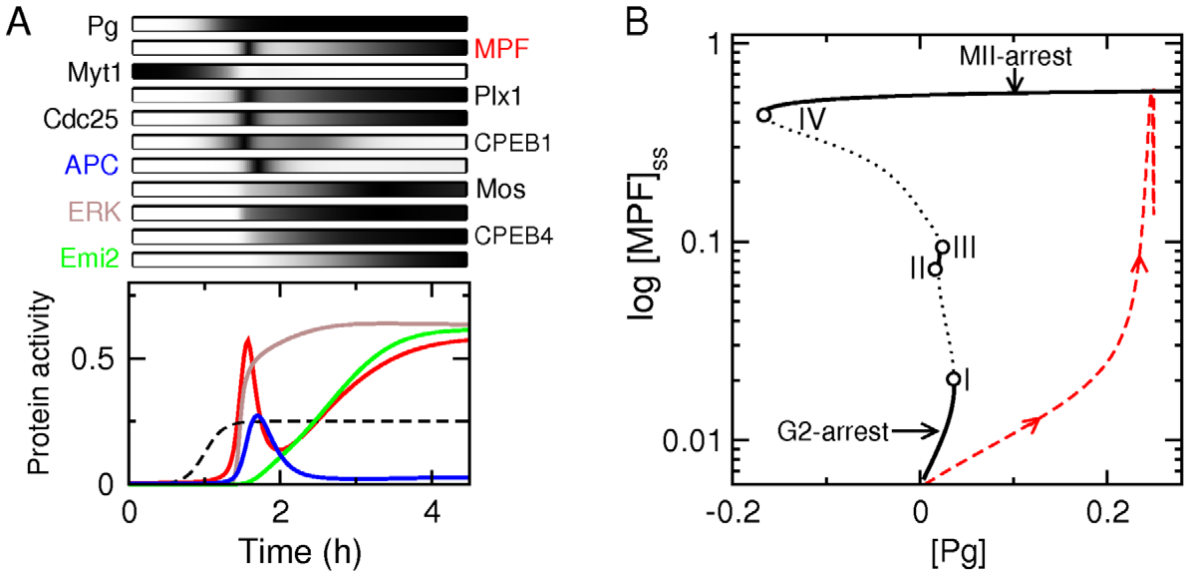
Complex bistable dynamics during meiotic maturation. (**A**) Time courses of various protein concentrations in response to a lasting progesterone pulse. We use the parameter set given by Table 2. In the top panel, time profiles are represented using a grayscale code which is normalized so that the maximal concentration of each protein corresponds to black. In the bottom panel, activities of MPF (*red*), ERK (*brown*), APC (*blue*) and Emi2 (*green*) are shown as functions of time, together with the constant progesterone signal (*dashed line*). (**B**) Bifurcation diagram showing the steady state of MPF activity as a function of progesterone level. Black solid and dotted lines are associated with stable and unstable equilibria, respectively. Circles indicates the occurence of a saddle-node bifurcation. The red dashed line shows the dynamic trajectory of MPF during progesterone-induced maturation.

Fig. 2A shows how the mathematical model responds to a constant exposure of progesterone. The numerical simulation reproduces the typical MPF temporal profile observed during meiotic maturation of *Xenopus* oocytes (compare Fig. 2A and Fig. 1B). In contrast with the non-monotonous time course of MPF, components of the MAPK pathway (Mos, MEK, ERK, Rsk) or of the autoamplification loop (Plx1, Cdc25, Myt1) exhibit a sharp activation (or inactivation for Myt1) followed by a plateau. Besides CPEB1, which is inactivated and degraded at the meiotic transition, APC is the only actor which exhibits a transient activation, following the MPF peak associated with anaphase events. Another key feature of the simulation is Emi2 activity rising only at the very end of meiosis I before reaching a plateau. As we shall see later, proper timing of Emi2 activation is crucial to allow MPF to reaccumulate after a full activation of APC. The activity profiles obtained in this simulation are fully consistent with experimental data collected for Mos, MAPK, Plx1, APC/Cdc27 or Emi2 during oocyte maturation of *Xenopus laevis* [4], [6], [7], [13], [34].

The one-parameter bifurcation diagram in Fig. 2B shows how the steady state value of MPF activity varies as a function of progesterone level. At least two stable solution branches coexist for some range of progesterone concentration, including the case of no progesterone. The coexistence of two (or more) stable solutions for the same parameter value is a phenomenon known as *bistability* (or *multistability*). Coexisting stable solution branches (*nodes*) are generally connected by an unstable branch solution (*saddle*), which acts as a separatrix between them. Stable and unstable branches connect at *saddle-node points*, where they annihilate together (the stable and unstable solution can be found on one side only of the saddle-node point). The lower branch (low MPF activity) corresponds to the prophase I-arrest state whereas the upper branch (high MPF activity) corresponds to the Metaphase II-arrest state. A sufficiently strong progesterone input, even transient, is therefore able to switch the cellular state from G2-arrest to metaphase-II arrest in an irreversible manner. An important feature of this bifurcation diagram is also the existence of four saddle-node bifurcation points (I, II, III, IV) instead of the two saddle-node points associated with the classic bistability scheme. The bifurcation point I controls the progesterone level required to destabilize the G2-arrested state and to trigger sharp MPF activation. The bifurcation point IV determines the stability of the metaphase II arrest characterized by high MPF activity. As long as this bifurcation point is associated with a negative value of progesterone signal, oocytes cannot leave the metaphase II state. If it is shifted to positive values of the progesterone signal by parameter changes, however, high MPF levels cannot be maintained, and the arrest state is unstable. The presence of the two additional saddle-node points II and III allows the slopes of the unstable solution branches originating from points IV and I to be largely independent, a feature which may persist even if the saddle-node points collide upon variation of another a parameter. This configuration effectively decouples the bifurcation points I and IV and allows them to be controlled separately, which will prove crucial in the following. The global structure of the bifurcation diagram of Figs. 2B, with its double bistability cycle, reflects in fact the coordinated actions of two bistable positive-feedback systems [18], [35], [36], which are relatively independent although one tends to activate the other and which will be identified in the next section. The non-monotonous behavior of MPF activity during the transition from the low activity state to the high activity state is not directly related to the structure of the bifurcation diagram. The fact that MPF activity rises, then decreases before increasing again is due to a negative feedback control based on the interaction between MPF and APC.

**Figure 3:**
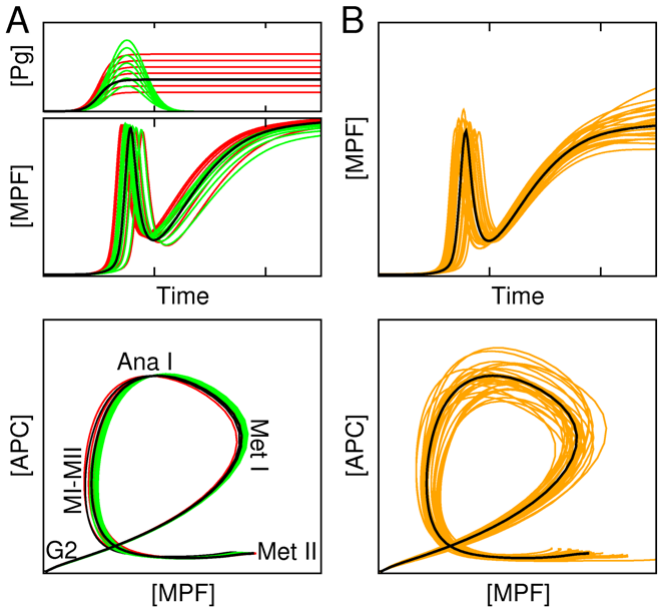
Robustness of meiotic dynamics to signal and kinetic parameter variability. Dynamical trajectories of [MPF] followed in time (top panels), and in state space ([MPF],[APC]) (bottom panels) during maturation in presence of two types of random variability. (**A**) Variations of progesterone input profile (green lines: pulse-like; red lines: step-like) and amplitude. (**B**) Variations of all kinetic parameter values with a 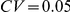 (orange lines). Thick black lines correspond to the control case depicted in Fig. 2. In bottom panel of (**A**) is indicated the maturation stage (MetI: metaphase of meiosis I, AnaI: anaphase of meiosis I; MI-MII: transition from meiosis I to meiosis II; MetII: metaphase of meisosis II) associated with distinct portion of the state-space trajectory.

This feedback-based bifurcation structure underlying the maturation dynamics is expected to provide robustness against environmental or intrinsic noises that could bias the trajectory toward inappropriate cellular states (e.g. G2-like, interphase-like, oscillations). Fig. 3 shows that such major disruption is very improbable. Indeed, the dynamical trajectory of MPF in state space starting from a G2-arrest state to a metaphase-II arrest state (bottom panel of Fig. 3A) is remarkably insensitive to changes in the progesterone input profile (top panel of Fig. 3A), with fluctuations mostly affecting the timing of maturation (middle panel of Fig. 3A). In particular, whether progesterone input is constant or transient has almost no effect on the dynamical trajectory of MPF, provided the input is sufficiently strong, which is consistent with experiments in which oocytes are either treated with transient or continuous exposure of progesterone [37]–[39]. It also confirms the result anticipated by the bifurcation diagram of Fig. 2B that oocyte meiotic maturation is indeed a bistable process in which the transition can be triggered by a transient perturbation. Similarly, the model also displays a robust behavior with respect to variability in kinetic rates since the qualitative structure of the trajectory remains unaffected when all kinetic parameter values are randomly changed with a coefficient of variation (CV) of 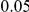 (Fig. 3B). For a CV of 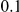, only a few cases display abnormal MPF profiles. However, when the CV of parameter changes is increased beyond the large value of 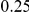, maturation failures occur in more than half of the trials.

The type of robustness oberved here emphasizes that achieving the appropriate sequence of decisions depends on the sequence of biochemical states traversed, not on the exact times at which they are reached. Thus, the state space trajectory is more relevant than time profiles. This robust dynamical behavior stems from the existence of an attracting slow manifold that canalizes the trajectory in state space.

### Parameter sensitivity analysis reveals a highly modular network

In the previous section, the bifurcation analysis revealed the existence of two positive-feedback control systems operating independently to sequentially drive the G2/M and meiotic transitions, in addition to the core MPF-APC negative feedback loop. To identify these two systems, numerical simulations and bifurcation analysis are here supplemented with a systematic parameter sensitivity analysis, which allows us to characterize the effect of each parameter on the maturation process.

We first focus on quantitative indicators of the MPF activity profile, which are the time of occurence 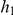 of the first MPF peak (signaling the G2/MI transition), as well as MPF levels 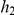 and 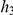 associated with the trough and the plateau of the MPF time course (signaling the MI/MII transition and the further metaphase II arrest). We measure their sensitivities to parameter variation as:
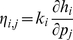
(1)where 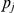 is the perturbed parameter and the 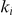 weigh the different indicators. As discussed above, G2 arrest and MII arrest are directly controlled by the saddle-node bifurcation points I and IV, respectively. Denoting by 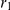 and 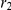 the progesterone thresholds associated with bifurcation points I and IV, respectively, the sensitivity of 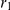 and 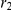 to parameter variation should also be a relevant parameter sensitivity measure of maturation dynamics. These sensitivities can be written as:
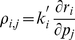
(2)For both types of sensitivities, it is useful to define normalized sensitivities, defined by 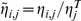 and 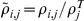 with 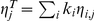 and 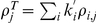. For example, a value of 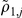 close to 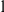 (resp., 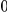) indicates that parameter 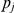 affects the progesterone threshold 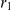 (resp., 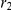) much more than 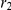 (resp., 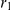).

**Figure 4:**
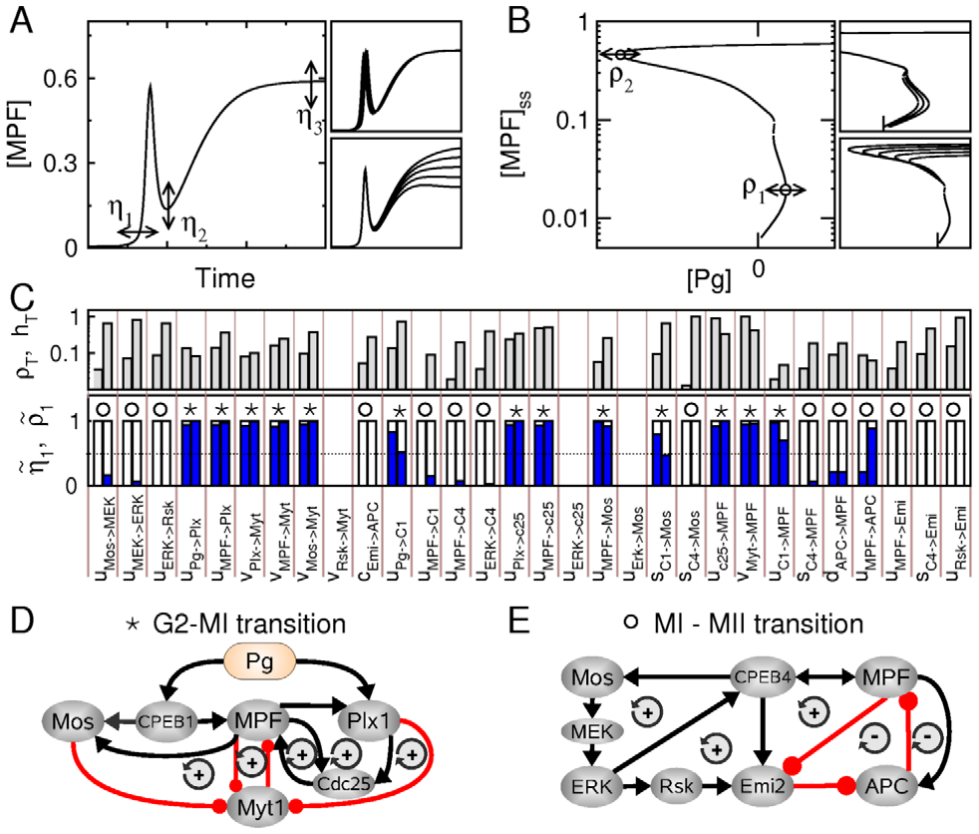
Identification of two network modules using parameter sensitivity analysis. (**A**) Schematic representation of the first sensitivity measure, where 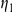, 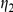 and 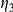 correspond to variations in timing of first MPF peak and in MPF levels at the MI/MII transition and at the metaphase II arrest, respectively, in response to parameter variations. Right-side panels show two examples where only 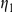 or 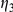 indicators is sensitive to the parameter changed. (**B**) Schematic representation of the second sensitivity measure, where 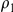 and 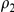 correspond to displacements of saddle points I and IV in the bifurcation diagram of Fig. 2B, in response to parameter variations. Right-side panels show two examples in which only one saddle-node bifurcation point is sensitive to the parameter changed. (**C**) Systematic calculation of normalized sensitivities for all interaction parameters of the model (see Eqs.1 and 2 with 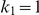, 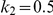, 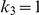, 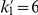 and 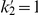). The top panel shows the total sensitivities 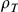 and 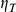. The bottom panel shows the normalized sensitivities 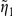 (left bar) and 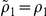 (right bar). Asterisks (resp., circles) indicate when 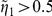 (resp., 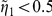). (**D**,**E**) The initial network can be redrawn as two networks that control different stages of maturation process, namely the G2/MI and MI/MII transitions. The 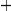 and 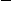 signs indicate the presence of positive and negative feedback loops, respectively.

These two complementary sensitivity measures are expected to indicate whether a given parameter tends to affect early or late stages of maturation as illustrated in the right panels of Figs. 4A and 4B. Fig. 4C provides a synthetic view of sensitivity values for all kinetic parameters that control interactions between two molecular actors and are therefore associated to a link in the network diagram of Fig. 1C. Interestingly, the values of sensitivities 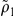 and 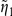 (and therefore 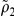 and 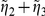) are seen to be highly correlated. The two sensitivities thus essentially provide the same information, confirming that dynamic response to progesterone signals is very much controlled by the bifurcation diagram. Noting that most values of the normalized sensitivities are either close to 0 or 1, a natural partition of kinetic parameters into two classes emerges, according to whether they preferentially control transition G2/MI (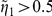) or the MI/MII transition (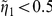). The classification so obtained allows us to disentangle the complex regulatory network shown in Fig. 1C by isolating two separate subnetwork module, such that all links in a module control the same transition. It is quite remarkable that most molecular actors appear in one module or the other but not in both, with the notable exception of Mos and MPF. Note that links corresponding to kinetic parameters with very small sensitivities have been neglected. Presumably, these molecular interactions have biological roles not directly related to maturation control or have a specific impact that could not be identified given the chosen model parameters.

The first circuit drawn in Fig. 4D displays a coherent feedback organization where only positive feedback loops are present. The post-translational interactions between Plx1, Cdc25, MPF and Myt1 constitute the core set of positive-feedback loops that contributes to the MPF autoamplification loop. Additional feedforward loops mediated by the activation of the translation machinery (i.e., CPEB) and positive-feedback loops mediated by the phosphorylation of Mos by MPF and of Myt1 by Mos are also involved in G2/MI transition. The architecture of the circuit controlling MI/MII transition (Fig. 4E) markedly differs from that of the meiotic resumption module. First, MPF is now regulated through CPEB-dependent synthesis and APC-dependent degradation, which control only its turnover. Second, this circuit relies on an antagonism between two negative feedback loops, where the direct interactions of MPF with APC and Emi2 promote its own inactivation through the degradation of its cyclin subunits, and a positive-feedback loop where MPF-dependent activations of MAPK and CPEB4 cooperate towards the accumulation and activation of Emi2, which itself opposes the APC-dependent degradation of MPF. This feedback antagonism results in an incoherent feedforward loop, which is key to the precise temporal gap between the G2/MI and MI/MII transitions.

### Simulated phenotypes of oocyte maturation defects

**Figure 5:**
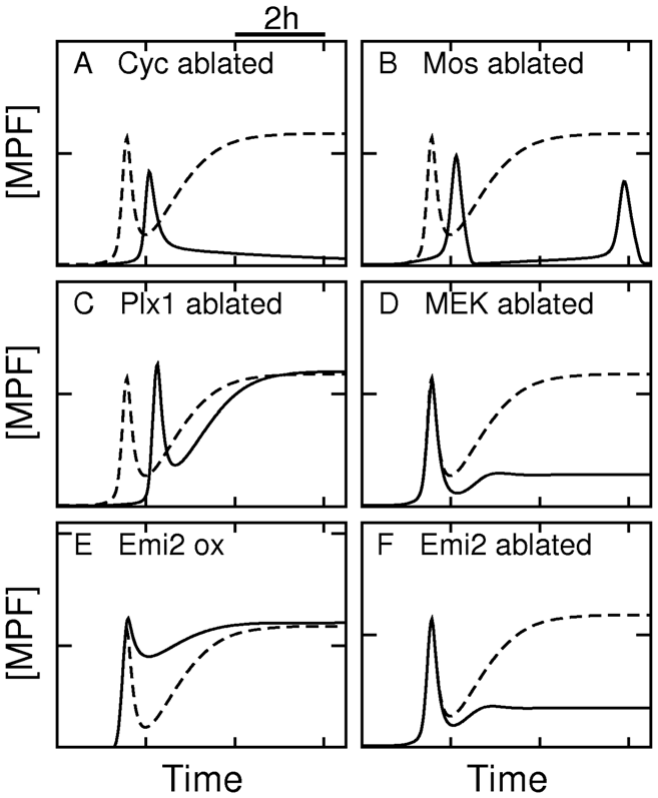
Simulated MPF time courses associated with meiotic maturation defects. Time course of MPF activity in normal condition (*dashed line*) and various altered conditions (*full line*). (**A**) Ablation of cyclin synthesis (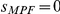). (**B**) Ablation of Mos synthesis (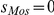). (**C**) Ablation of Plx1 activity 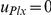. (**D**) Ablation of MEK activity (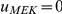). (**E**) Overexpression of Emi2 (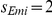). (**F**) Ablation of Emi2 synthesis (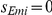).

Importantly, our model accounts not only for the main features of meiotic maturation in wild-type eggs, but also of phenotypes of eggs treated by antisense oligonucleotides-based strategies or by chemical inhibitors. Our simulations of these phenotypes are summarized in Fig. 5 and Table 1.

**Table 1:** Examples of oocyte maturation defect phenotypes.

Protocol	Phenotype	References	Model parameters	Figures
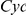 ablated	MI-entry delay	[8]	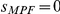	Fig. 5A
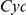 ablated	MII-entry failure	[4]	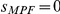	Fig. 5A
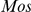 ablated	MI-entry delay	[5], [8]	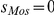	Fig. 5B
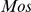 ablated	Oscillations	[7]	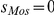	Fig. 5B
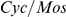 ablated	MI-entry failure	[8]	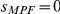 , 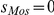	Not shown
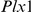 ablated	MI-entry delay/failure	[22], [40]	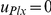	Fig. 5C
U0126( 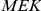 inhibitor)	MI/MII failure	[5], [6]	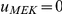	Fig. 5D
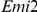 overexpressed	MI arrest	[11]	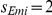	Fig. 5E
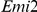 ablated	MII-entry failure	[10], [20]	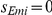	Fig. 5F

Pharmacological or antisense treatments impacting the activity of several specific proteins lead to various sorts of maturation defect phenotypes as reported in the literature. B-type cyclin is abbreviated as *Cyc*. Changing specific parameters of the model allows to simulate these phenotypes, which is also shown in Fig. 5.

To identify the role of protein synthesis in the initiation of *Xenopus* oocytes maturation, experiments have been performed to inhibit cyclin B or/and Mos synthesis using antisense oligonucleotides [8]. They showed that ablation of either Mos or cyclin B alone does not prevent maturation initiation yet induces significant delays, whereas combined ablation impairs initiation. Our model is able to reproduce such delays in the absence of cyclin or Mos synthesis (Fig. 5A and B), reflecting the existence of cooperative mechanisms between translational and post-translational controls during meiotic initiation (e.g., Mos synthesis and Mos-dependent inactivation of Myt1). In addition, oocytes where cyclin B is disabled by antisense strategies fail to reaccumulate MPF at MI/MII transition [4]. This is also observed in simulations (Fig. 5A) where, after the post-translational activation of preMPF by the Plx1 pathway, depletion of preMPF and degradation of active MPF by APC are not counterbalanced by the synthesis of new cyclins, thereby precluding MPF reaccumulation. Meiotic transition also fails in Mos-ablated oocytes, due to the absence of MAPK activation [7], with the possibility however to form a transitory interphase nucleus after completion of meiosis I and to reactivate MPF so as to mimic the mitotic cell cycle of early embryos [7]. In numerical simulations, oocytes lacking Mos are indeed unable to transit appropriately to MII. However, an oscillatory pattern of MPF activity may be also observed although it is highly sensitive to model parameters (Fig. 5B). Besides the defects for maturation initiation associated with inhibition of protein synthesis, disruption of the progesterone-dependent Plx1 activation also significantly delays meiotic resumption in progesterone-treated oocyte [22], [40], which is reported as well in numerical simulations (Fig. 5C). Note that any combination of the disruption of cyclin synthesis, Mos synthesis and Plx1 activation leads in model simulation to maturation initiation failures (result not shown), emphasizing the synergistic role of multiple translational and post-translational mechanisms.

Inhibition of MAPK activation in oocytes can also be achieved using MEK inhibitor U0126 [5], [6], [41]. In U0126-treated oocytes, MAPK inactivation prevented cyclin B reaccumulation after MI, by allowing APC-mediated degradation similarly as in the case of Emi2 ablation [10]. In simulations (Fig. 5D), MPF concentration does not vanish as in the case of inhibition of cyclin synthesis but remains at an intermediate level as is observed in experiments [5], [6]. Simulations do not reproduce the delay observed in these experiments, which can be due to our model not taking into account the regulation of cyclin B synthesis by MAPK as has been reported by Abrieu *et al* [42]. In addition, chemical inhibitor U0126 might target other translational regulators besides MEK1, and such non-specific effects may account for discrepancies in the observations made when Mos is ablated.

Experiments inducing deletion or overexpression of Emi2 demonstrate the crucial role of this protein in meiotic transition. Ectopic expression of Emi2 at physiological MII levels can arrest maturing oocytes at metaphase I [11], which is easily explained by the fact that Emi2 counteracts APC activity and subsequently cyclin degradation, maintaining a sustained MPF activity (Fig. 5E). Conversely, our simulations also reproduce the effect of inhibiting Emi2 synthesis (Fig. 5F), which leads to complete and rapid degradation of cyclin B at MI exit, causing an inappropriate exit into interphase and a failure to reaccumulate cyclin B [10], [11], [13]. These experiments and simulations showing maturation failure for overexpression or deletion of Emi2 strongly support that a strict temporal control over Emi2 levels is critical for a reliable MI/MII transition.

## Discussion

In this work we have designed and analyzed a detailed mathematical model describing the meiotic maturation process that begins when an oocyte is released from G2-arrest and terminates when it is arrested in metaphase of meiosis II. We have unveiled how maturation is driven by a highly dynamic coordination between the core mitotic oscillator, based essentially on MPF, Cdc25 and APC, and the MAPK signaling pathway, which are both stimulated by the same extracellular signal. Although the model does not incorporate several regulatory schemes discovered recently [34], [43], it appears to be sufficiently detailed to gain insight into the essential features of maturation and to discriminate the roles of different regulatory motifs. It can therefore serve as a solid basis for further explorations.

Resumption of meiosis requires MPF activation, which is potentially mediated by a multiplicity of pathways, including Plx1-dependent changes of Myt1-Cdc25 balance as well as Mos-dependent inhibition of Myt1 and Cyclin synthesis. Inhibiting one of these pathways in the model delays or compromises maturation initiation, as in experiments [8], [40]. This suggests redundant and cooperative roles between these various translational and post-translational MPF activation schemes. However, model parameters can be adjusted so that cyclin B synthesis or Mos activation occur after GVBD and would therefore be fully dispensable for MPF activation as it is observed in various organisms [42]. The role of MAPK is more pronounced at the meiotic transition where it leads to the Rsk-dependent activation of Emi2 required for MII entry and metaphase II arrest [10], [20]. Simultaneous activation of MAPK and MPF raised nevertheless intriguing questions (Wu and Kornbluth, 2008): why do eggs arrest at MII but not at MI? What causes the delayed and sharp activation of Emi2? Our model reconciles different views on these questions by showing that both the late translational control by CPEB4 (Igea and Mendez, 2010) and the temporally-controlled antagonistic roles of MPF and Rsk in stabilizing Emi2 [13] contribute to this delay.

It is worth mentioning that our modeling analysis does not capture the role of the few links that couple downstream effectors of MAPK - essentially ERK and Rsk - with components Cdc25 and Myt1 of the autoamplification loop [30], [31], [33], [44]. It remains unclear whether and how these MPF-activating pathways contribute to G2/M transition and to meiotic transition. A controversial hypothesis is the existence of a transient activation of MAPK or/and Rsk shortly after progesterone injection, stimulating MPF activation [33], [45], [46], which could be easily incorporated into models if needed. Another possibility is that these regulations also play a role in consolidating Myt1 inactivation and Cdc25 activation at meiotic transition to compensate the transient MPF activity decrease, an hypothesis that needs to be further tested in both models and experiments.

### Feedback principles underlying meiotic decisions

**Figure 6:**
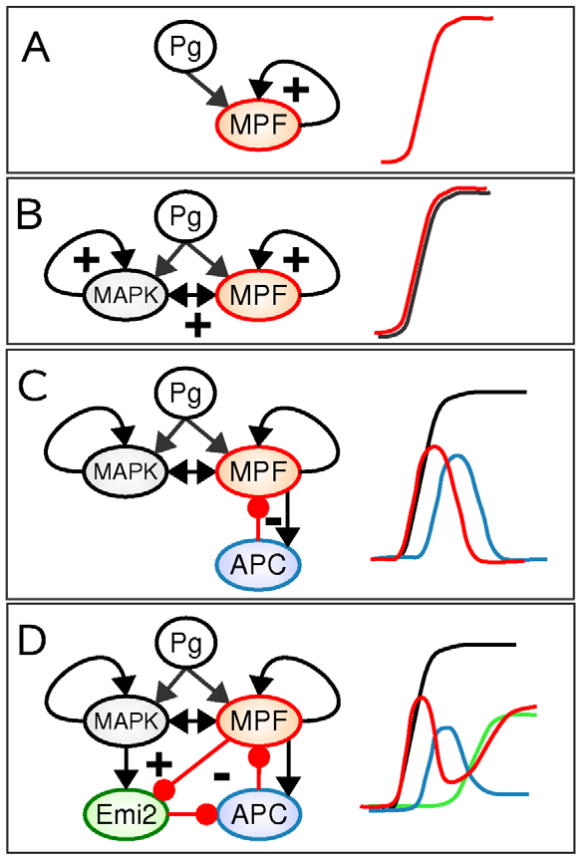
Feedback design principles of oocyte meiotic maturation. (**A**)The auto-amplification positive-feedback loop switches on MPF activity (Red). (**B**) Coupled positive-feedback loops ensure a coherent switch of the MPF and MAPK activities (black). (**C**) The negative-feedback loop linking MPF and APC (blue) triggers a transient decrease of MPF activity that does not impact the high MAPK activities maintained by independent positive-feedback loops. (**D**) Delayed activation of a positive-feedback loop mediated by Emi2 (green) antagonizes the negative-feedback loop, so as to fully reactivate MPF.

The availability of a regulatory network model that qualitatively reproduces a broad spectrum of experimental data allows us to investigate the design principles that underlie a reliable maturation process. Bifurcation and sensitivity analyses of the model unveiled the existence of two independent subcircuits where feedback loops are subtly interlocked so as to achieve two coordinated but separable transitions (see Fig. 6).

The first transition, meiotic resumption, relies on a circuit that involves several signaling pathways and positive-feedback loops. This module is organized around the core autoamplification loop which includes MPF, Myt1, Cdc25 and Plx1 and drives the sharp post-translational activation of MPF associated with G2/MI transition (Fig. 6A). This loop is supplemented with other positive-feedback loops and coherent feedforward loops featuring CPEB1 and Mos, which ensures a simultaneous activation of MPF, the Mos/MAPK pathway and the translational machinery (Fig. 6B). The role of these combined positive-feedback motifs in the MPF and MAPK modules is not related to robustness against noise [47], activation threshold tuning [48] or multistability [18], [35], but is rather aimed to induce and sustain high MAPK activity throughout maturation, independently of the MPF activity level which decreases due to the APC-dependent degradation of cyclin subunits at the end of the first meiosis (Fig. 6C).

The second transition from meiosis I to meiosis II indeed requires high MAPK levels to promote MPF stabilization. Late reactivation of MPF is driven by a delayed positive-feedback loop involving Emi2 that counteracts the negative feedback mediated by APC. Delayed activation of Emi2 is itself the result of the incoherent feedforward loop in which MPF both activates and inactivates Emi2 (Fig. 6D). This sophisticated regulatory scheme provides an interesting example of how the combination between positive and negative feedback loops gives rise to complex dynamics such as non-monotonous bistable behaviors, besides those that have already been studied in the context of oscillatory, excitable and bistable dynamics [36], [49], [50].

### Conclusion

Meiotic maturation poses a difficult challenge to oocyte cells. A single transient signal must be followed by a coordinated sequence of two crucial and distinct decisions, MI entry and MI/MII transition, which both require a sharp MPF activation. Our findings reveal the sophisticated molecular network mechanisms that provide an original solution to this problem. Firstly, like in other biological decision-making processes, the two main meiotic decisions rely on two distinct positive-feedback-based circuits, each of which combining multiple loops so as to create sharp and robust transitions. Secondly, interference and retroactivity between the two decision circuits are minimized by using separate and partly independent regulatory schemes based on post-translational modifications and protein turnover control, respectively. Lastly, the coordination of the decision systems is mediated by the existence of a negative feedback loop and an incoherent feedfoward loops, which are known to be efficient for scheduling temporal gaps between successive decisions [51], [52]. Thanks to this specific regulatory and feedback architecture, a transient signal can trigger complex dynamical and phenotypical trajectories which are attracted by a one-dimensional slow manifold and follow it throughout maturation. This dynamical process is reminiscent of the phenomenon of canalization during multicellular development [53]. Overall, this encourages further efforts to decipher the dynamical behavior of molecular networks with complex feedback and feedforward topology, especially when they combine oscillatory and irreversible behaviors, as occuring during meiotic maturation.

## Methods

### Mathematical modeling of the maturation regulatory network

The mathematical model for the maturation regulation network is based on the molecular interactions reported in *Xenopus laevis* between the 12 proteins CPEB1 (abbreviation C1), CPEB4 (abbr. C4), MPF, Cdc25 (abbr. C25), Myt1 (abbr. Myt), APC, Mos, MEK, ERK, Rsk, Plx1 (abbr. Plx) and Emi2 (abbr. Emi). We assume that the activity of each protein in the list above can be post-translationally regulated, typically through phosphorylation, such that they can be either in activated or inactivated forms. The 12 molecular actors can be distinguished according to whether their total concentration is also regulated (class I: Mos, MPF, APC, Emi, C1, C4) or can be considered as constant on the time scale of maturation (class II: Plx, C25, Myt, MEK, ERK, Rsk).

For class-I proteins, the concentrations of the active and inactive proteins evolve in time according to a set of differential equations (Table 2). where 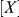 and 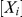 denote the concentration of the active or inactive forms of protein 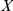, whereas 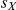, 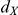, 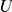 and 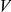 denote, respectively, the synthesis, degradation, activation and inactivation rates of these proteins. We assume Michaelis-Menten kinetics for activating and inactivating reactions where 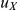 and 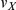 are the maximum rate of the reactions (Table 2). Only Emi2 is assumed to have more than two states: (i) partially activated when unphosphorylated with a dephosphorylation reaction rate 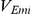; (ii) inactivated through phosphorylation by Rsk with a reaction rate 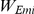; (iii) fully-activated through phosphorylation by Rsk of unphosphorylated or MPF-phosphorylated forms, with a reaction rate 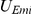. The concentrations of these three forms are denoted by 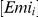, 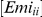 and 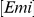, respectively. The assumption that the total concentrations of class-II proteins remain relatively stable throughout the maturation process allows us to use the quasi-steady state approximation. Moreover, if phosphorylation and dephosphorylation reactions operate in the linear regime, the steady-state concentrations can be obtained as a function of the total concentration (normalized here to 1) and of their maximum activating and inactivating reaction rates 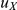 and 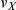. These expressions depend on whether activation is achieved through a one-step phosphorylation (C25, Myt, Plx) or a two-step phosphorylation (MEK, ERK, Rsk) (Table 2).

**Table 2:** Equations of the model.

**Differential equations**
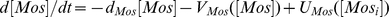
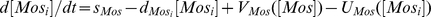
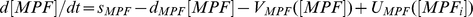
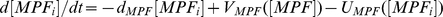
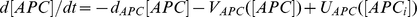
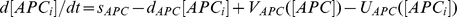
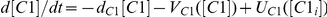
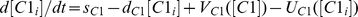
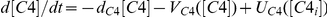
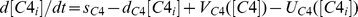
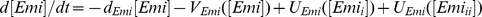
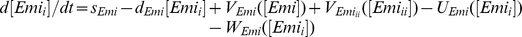
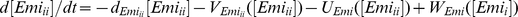
**Michaelis-Menten kinetics**
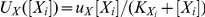
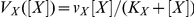
**Steady-state concentrations**
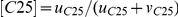
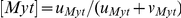
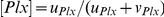
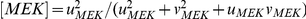
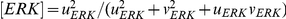
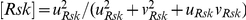
**Kinetic reaction rates**
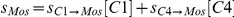
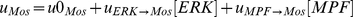
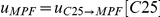
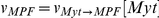
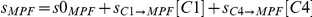
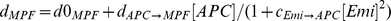
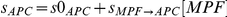
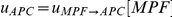
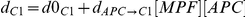
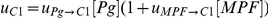
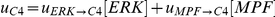
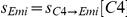
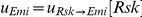
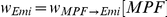
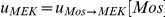
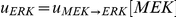
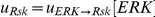
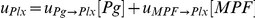
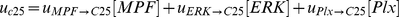
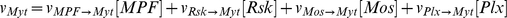

Dynamic and steady-state equations associated with the network shown in Fig. 1C (see also Methods).

**Figure 7:**
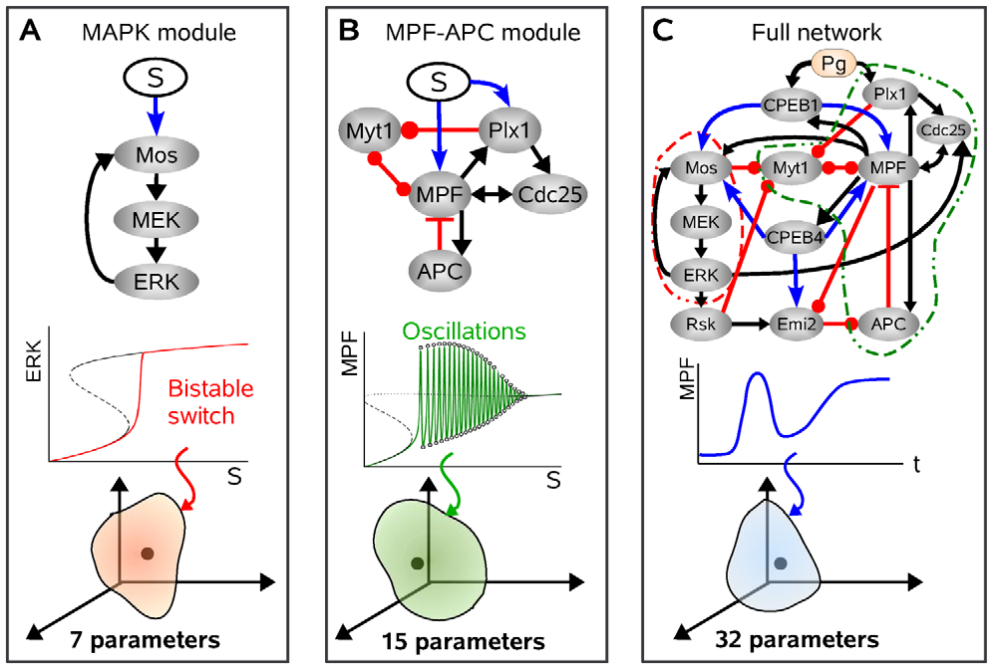
Strategy for the adjustment of model parameters. (**A**) The constraint that the MAPK module (upper panel) must behave as a bistable switch (example of a bifurcation diagram in middle panel) allows one to determine a parameter domain in a 7-dimensional parameter space (bottom panel). An arbitrary parameter set is chosen within this domain. (**B**) The constraint that the MPF-APC module (upper panel) must behave as an oscillator under constant stimulation or be excitable upon a transient stimulation, which is associated with a saddle-node bifurcation on an invariant circle (example of a bifurcation diagram in middle panel), allows one to determine a parameter domain in a 15-dimensional parameter space (bottom panel). An arbitrary parameter set is chosen within this domain. (**C**) The constraint that the whole network (upper panel) must display a maturation behavior associated with a specific time course of its components (MPF time course as an example in middle panel) allows one to determine a parameter domain in the remaining 32-dimensional parameter space (bottom panel). An arbitrary parameter set is chosen within this domain.

The kinetic rates 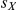, 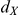, 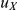 and 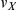 that appear in both differential equations and steady-state concentrations can be either considered as constant parameters on the time scale of maturation process or as time-dependent variable as they may depend on the concentration of other dynamic regulators of the maturation process. Such dependence is depicted as links in the network representation of Fig. 7 is given in Table 2.

### Choice of model parameters for reaction kinetics

The model contains a large number of kinetic parameters (73), which, for the most, have not been estimated experimentally so far. A preliminary step toward the adjustment of parameters is to reduce their number. To account for the dynamical features of the maturation process, the mathematical model only needs to describe the evolution of protein concentrations relative to each other. We can therefore normalize protein concentrations. First, the total concentration 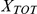 of class-II proteins is normalized to 1. Second, only the relative value between activation rates and inactivation rates are relevant for class-II proteins, such that we can introduce a free parameter 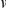 which determines their absolute value. Third, the Michaelis constants for all activation and inactivation processes of class-I proteins are set to 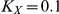. Actual values of the concentrations can always be recovered by scaling the variables appropriately, keeping in mind that the present modeling study focuses on the temporal profile of protein activity rather than quantitative predictions. The normalization procedure can reduce the number of parameter to 54. The other parameters used in this study (Table 3) have been selected in a semi-arbitrary manner constrained by qualitative fitting of the time course of several components in various contexts. The kinetic parameters for the MAPK pathway have been adjusted to display the classic bistable behavior of this cascade (Fig. 7A). The kinetic parameters for Cdc25, Myt1, MPF, APC and their respective interactions have been adjusted to produce an excitable or oscillatory behavior commonly associated with a specific underlying bifurcation structure of the dynamics: a saddle node bifurcation on an invariant circle (Fig. 7B). Finally, the kinetic parameters coupling these two modules between themselves and to the input signal have been adjusted to match the temporal profile of MPF activity that is typically observed in various experimental prototocols (Fig. 7C, see also Fig. 1B and 5).

**Table 3:** Model parameter values.

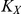	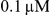	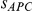	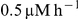	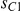	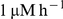
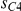	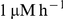	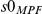	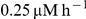	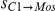	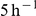
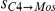	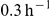	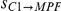	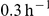	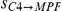	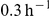
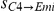	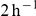	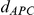	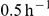	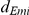	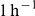
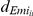	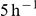	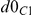	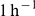	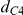	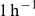
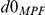	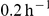	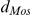	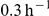	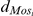	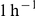
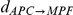	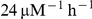	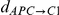	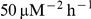	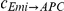	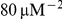
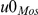	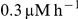	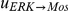	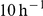	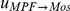	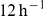
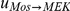	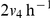	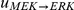	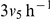	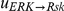	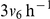
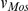	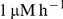	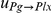	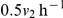	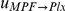	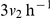
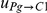	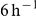	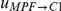	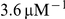	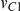	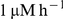
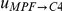	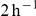	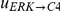	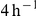	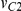	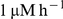
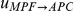	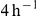	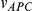	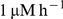	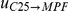	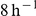
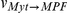	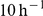	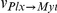	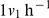	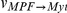	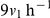
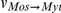	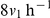	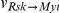	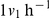	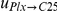	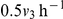
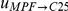	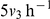	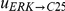	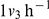	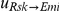	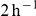
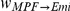	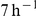	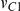	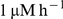	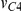	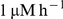
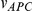	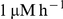	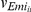	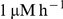	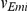	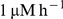
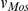	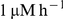	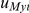	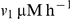	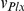	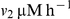
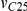	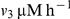	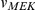	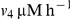	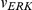	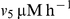

Parameter values result from adjusting qualitatively the behavior of various configurations of the model to experimental data (see Methods). Note that the actual value of 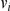 can be arbitrary chosen.
